# Towards a Multimodal Model of Cognitive Workload Through Synchronous Optical Brain Imaging and Eye Tracking Measures

**DOI:** 10.3389/fnhum.2019.00375

**Published:** 2019-10-23

**Authors:** Erdinç İşbilir, Murat Perit Çakır, Cengiz Acartürk, Ali Şimşek Tekerek

**Affiliations:** ^1^Advanced Technologies Directorate, Guidance and Photonics Division, Roketsan Missiles Industries Inc., Ankara, Turkey; ^2^Department of Cognitive Science, Informatics Institute, Middle East Technical University, Ankara, Turkey

**Keywords:** neuroergonomics, fNIRS, eye tracking, human machine interface, cognitive workload

## Abstract

Recent advances in neuroimaging technologies have rendered multimodal analysis of operators’ cognitive processes in complex task settings and environments increasingly more practical. In this exploratory study, we utilized optical brain imaging and mobile eye tracking technologies to investigate the behavioral and neurophysiological differences among expert and novice operators while they operated a human-machine interface in normal and adverse conditions. In congruence with related work, we observed that experts tended to have lower prefrontal oxygenation and exhibit gaze patterns that are better aligned with the optimal task sequence with shorter fixation durations as compared to novices. These trends reached statistical significance only in the adverse condition where the operators were prompted with an unexpected error message. Comparisons between hemodynamic and gaze measures before and after the error message indicated that experts’ neurophysiological response to the error involved a systematic increase in bilateral dorsolateral prefrontal cortex (dlPFC) activity accompanied with an increase in fixation durations, which suggests a shift in their attentional state, possibly from routine process execution to problem detection and resolution. The novices’ response was not as strong as that of experts, including a slight increase only in the left dlPFC with a decreasing trend in fixation durations, which is indicative of visual search behavior for possible cues to make sense of the unanticipated situation. A linear discriminant analysis model capitalizing on the covariance structure among hemodynamic and eye movement measures could distinguish experts from novices with 91% accuracy. Despite the small sample size, the performance of the linear discriminant analysis combining eye fixation and dorsolateral oxygenation measures before and after an unexpected event suggests that multimodal approaches may be fruitful for distinguishing novice and expert performance in similar neuroergonomic applications in the field.

## Introduction

Understanding the neural underpinnings of complex cognitive tasks in the context of safety-critical settings is a key objective for neuroergonomics research (Parasuraman, 2011; Parasuraman et al., 2012; Mehta and Parasuraman, 2013). Operators’ effective utilization of human-machine interfaces in such settings depends on interface design considerations, as well as the operators’ level of expertise in carrying out key tasks *via* the interface. Cognitive or mental workload is an essential determinant of operator performance that needs to be optimized through effective training and user-centered design practices. Cognitive workload addresses the limited capacity of the human brain for processing information demanded by the task at hand (Wickens and McCarley, 2007). Decrements in performance, as evidenced in missed responses or delayed response times, are typically observed when operators are subjected to cognitive workload beyond the maximum information processing capacity of their brains (Hancock and Parasuraman, 1992; Wickens et al., 2013). In other words, cognitive workload is an essential determinant of cognitive performance. However, these limits are subject to change due to the development of expertise during training, and design elements that promote or hinder the utilization of the system’s affordances, which altogether make their assessment a challenging issue (Fairclough et al., 2005; Ullén et al., 2018).

Due to its essential role in cognitive performance, developing operational measures for monitoring cognitive load is an active area of investigation in neuroergonomics. Such measures have been traditionally monitored offline through subjective assessments with the help of instruments such as NASA-TLX (Hart and Staveland, 1988; Hill et al., 1992). Despite their convenience for administering these instruments in various real-life and experimental task settings, the subjective nature of the assessments obtained and the difficulties participants have in providing verbal accounts of their actions motivated the need for more objective means for measurement. Recent studies in psychophysiology and neuroergonomics have expanded on self-evaluation based methods by focusing on neurophysiological measurements targeting key components of the central and the peripheral nervous system, as well as the cardiovascular system (for extensive reviews, see Fairclough, 2009; Borghini et al., 2014; Lim et al., 2018; Charles and Nixon, 2019).

Increase in operator workload is often associated with increase in heart rate, heart rate variability, respiration rate, blood pressure and galvanic skin conductance (Boucsein, 2012; Borghini et al., 2014; Durantin et al., 2014). However, the existence of other sources of variability competing with mental workload such as anxiety, muscle fatigue and room temperature present challenges for mental workload estimation methods based on these measures. Related work focusing on ocular correlates of cognitive workload tend to employ eye trackers and electrooculograms (EOGs) to relate measures such as fixation duration, saccadic amplitude, gaze entropy, pupil dilation, PERCLOS (the percentage of time that the eyelid covers 80% or more of the pupil), gaze blink frequency and latency with changes in workload. In particular, increase in cognitive workload is associated with increases in fixation duration, pupil dilation and blink latency, as well as decreases in blink duration, PERCLOS and gaze variability; whereas investigations on blink rate have produced mixed results (Marquart et al., 2015; Diaz-Piedra et al., 2019). External sources of variability such as changes in lighting conditions or the anatomic variation of the eyelids can influence the eye tracking, pupillometry and EOG measurements (Holmqvist et al., 2011).

Cognitive workload assessment approaches based on brain physiology mainly focus on electroencephalography (EEG) and functional near-infrared spectroscopy (fNIRS), due to their portability and flexibility that make them suitable for in-the-field neuroimaging studies. Studies on neural correlates of mental workload with EEG tend to focus on changes in the EEG power spectra, where an increase in workload is typically associated with an increase in the theta band power, especially at the top- and mid-central electrodes (Borghini et al., 2017). Since EEG monitors changes in electric potentials originated in the brain *via* electrodes located over the scalp, it offers high temporal resolution. However, due to the complexities involved with the propagation of electric potentials within neural tissue, achieving good spatial resolution requires high-density EEG recording setups with sufficient number of electrodes (Gevins and Smith, 2008). The presence of artifacts such as eye movements, muscle contractions and heart-beat, the need for ensuring low impedance levels for suitable signal-to-noise ratio, and the need for using gels or liquids to improve conductance are other challenges involved with the use of EEG for monitoring workload in the field (Dussault et al., 2005; Borghini et al., 2011; Borghini et al., 2014).

fNIRS monitors the hemodynamic changes over the cortex by using specific wavelengths of infra-red light that are known to interact with oxygenated and deoxygenated hemoglobin in the capillary beds (Obrig et al., 2000). fNIRS is a neuroimaging modality that enables continuous, noninvasive, and portable monitoring of changes in blood oxygenation and blood volume related to human brain function (Ayaz et al., 2013). fNIRS is not only non-invasive, safe, affordable and portable, it also provides a balance between temporal and spatial resolution which makes it a viable option for *in-the field* neuroimaging. Neuroergonomics studies employing the fNIRS modality reported a strong relationship between increasing bilateral dorsolateral prefrontal cortex (dlPFC) activity and systematic increases in cognitive workload (Izzetoglu et al., 2003; Gateau et al., 2015; Çakır et al., 2016). In combination with performance measures, this relationship could be fruitfully employed to evaluate alternative human-machine interaction designs (Menda et al., 2011; Ayaz et al., 2013) and to monitor expertise development as a function of the systematic decrease in prefrontal activity accompanied by improvements in performance in time (Ayaz et al., 2012). Moreover, the way infrared photons migrate in the neural tissue allow strategic placement of the sensor to target specific cortical regions close to the surface with a reasonable spatial resolution (Obrig et al., 2000). However, the latency involved with the hemodynamic response in reaction to neural activity is a limiting factor on the temporal resolution of the fNIRS methodology (Gratton and Fabiani, 2008).

To sum up, each modality offers specific advantages and limitations in terms of quantifying a complex construct such as mental workload due to the differences in their physical working principles and the relationship between the monitored biological processes to cognitive phenomena (Fairclough, 2009). Multimodal neurophysiological models that can bring the advantages of each modality together in contextually sensitive ways is an emerging necessity in neuroergonomics research (Fairclough et al., 2005). Existing efforts include simultaneous use of EEG and fNIRS (Aghajani et al., 2017; Liu et al., 2017a,b), as well as other physiological sensors (De Rivecourt et al., 2008; Gateau et al., 2015), and reported superior workload classification performance during working memory tests such as n-back as compared to predictions obtained from any single modality. However, conducting multimodal data recording and analysis brings significant challenges, such as sensor placement and shielding for reliable data acquisition, minimization of interference among the chosen modalities, time-synchronization of data streams, obtaining measurements without interfering with the operators’ normal task execution, and most importantly, finding appropriate means to integrate the data and insights obtained from the chosen modalities.

### Cognitive Workload and Expertise

In addition to the challenges involved with multimodal investigation of cognitive workload, another key source of variability in its assessment is due to individual differences in expertise with regards to the task at hand, which further complicates the development of metrics and/or thresholds for mental workload classification (Charles and Nixon, 2019). Related studies in cognitive neuroscience comparing experts and novices have identified both structural and functional changes due to expertise development, including the growth of gray and white matter in task-relevant cortical locations, as well as the reduction of activity in prefrontal and parietal cortex involved in domain-general cognitive control (Hill and Schneider, 2006; Bilalić et al., 2011; Bilalić, 2017; Bilalić and Campitelli, 2018). In particular, related work in expertise and distinguishing expertise levels in laparoscopic surgery skills showed that in contrast to novices experts exhibit greater performance under time pressure accompanied with significantly lower PFC activations and significantly higher primary motor area and supplementary motor area (SMA) activations, which are regions associated with bimanual motor dexterity performance (Modi et al., 2018; Nemani et al., 2018). Moreover, related work in gaze measures of expertise suggest that experts tend to exhibit more well-defined scan-paths towards task-relevant locations in the visual field, which is manifested in lower gaze entropy, lower fixation durations and shorter saccadic amplitudes (McCarley and Kramer, 2006). In a related research on expert and novice endoscopists’ gaze patterns, the results showed that there are distinct gaze patterns that are associated with expert behavior (Lami et al., 2018). However, such differences can be highly contextual with respect to the specific task domain and the way expertly conduct is characterized in that domain (Hodges et al., 2006). Therefore, a broader view of expertise is needed to make sense of the underlying causes of the differences obtained when experts and novices are contrasted along various neurophysiological measures.

The body of research in psychology and cognitive neuroscience of expertise suggest that most forms of expertise in domains such as sports, chess, math, dance and music entail: (i) a process of automation; (ii) reorganization of memory resources; and (iii) sensorimotor adaptations (Ullén et al., 2018). Sensorimotor adaptations involve changes due to extensive motor practice, such as the development of complex reflexes and sensitivities in sports like tennis, and the corresponding changes in the somatosensory, premotor and motor cortices such as the enlargement of the representations of the fingers of the left hand for string players (Elbert et al., 1995). Automation refers to a change from a more effortful, flexible, deliberate mode of carrying out tasks that relies on attention and working memory resources towards a less effortful, reflex like, low effort, difficult to modify mode of conduct that requires less attentional resources due to training and experience (Hill and Schneider, 2006). Since experts require little explicit control, surplus cognitive resources can be allocated for strategic decision making and planning. Together with before-mentioned sensorimotor adaptations presumably occurring in the premotor cortex guiding the eye movements, the notion of automation could also be manifested in the scan-paths of experts guiding the expert’s motor actions (Hodges et al., 2006). In neuroimaging, such adaptations are usually manifested as a decrease in the fronto-parietal network that is associated with deliberate guidance of attention (Duncan, 2010, 2013).

Experts also tend to exhibit superior recognition of complex sensory stimuli, which is claimed to be facilitated by mechanisms of efficient retrieval of relevant domain-specific information from long term memory into working memory during performance (Guida et al., 2012, 2013). According to this view, with developing expertise specialized long-term memory elements with complex knowledge structures called chunks are formed in the medial temporal lobe, which can be accessed by working memory, effectively increasing the information processing capacity of the expert. As the knowledge elements get bound into chunks within working memory, an increase in medial temporal lobe activation is accompanied by decreasing activations in prefrontal and parietal areas that are typically associated with memory retrieval and working memory management (Just and Carpenter, 1992). In terms of eye movement metrics, changes in the memory organization may also influence the way the visual information is sampled as perceptual input. This could be partly the underlying reason for the experts’ lower number of fixations and shorter fixation durations, as they can pick up and interpret the visual cues in a more efficient manner according to the related work in psychology of expertise (Hodges et al., 2006).

Overall, the growing body of literature in neuroergonomics have utilized increasingly portable/wearable neurophysiological measurement modalities in various work settings. However, the need for applying these methods and insights in multitude of field settings to test their robustness and assess their degree of domain specificity are still important considerations for the burgeoning field of neuroergonomics. To that end, in this study, we explored the simultaneous use of fNIRS and eye tracking in a human-machine interaction scenario involving a military land platform. The goal of the study is to observe in what ways experts and novices differ in terms of their prefrontal hemodynamic responses and eye movement patterns during regular and adverse task conditions in the field. The study aimed to investigate to what extent expertise levels modulated some of the expected neurophysiological correlates of cognitive workload as manifested in fNIRS and eye tracking modalities in a realistic setting with professional operators. Finally, the study explored a possible multivariate integration of prefrontal hemodynamics and gaze data to support expert vs. novice distinction.

### Materials and Methods

#### Participants

The study included a human-machine interaction scenario where the participants (Mean_Age_ = 28.9, SD = 3.6) were asked to operate a training version of a military land platform by using a computer-based control interface. The experimental procedures were conducted in the field with 14 professional, real-life operators, eight of whom were identified as experts due to their prior training on the platform, whereas six were novices/beginners who were on their first training day. All operators had prior experience in other comparable military platforms. Those operators who were considered as novices were all using the interface for the first time after receiving a formal presentation of the basic features of the system in a classroom setting. The experiment was conducted when this cohort moved on to the vehicles to perform hands-on exercises. An independent *t*-test showed that the age difference between the novices (Mean = 27.3, SD = 2.1) and the experts (Mean = 30.5, SD = 3.9) was not significant, *t*_(12)_ = −1.76, *p* > 0.05. Participants were informed prior to the study that the main goal of the experiment was to perform a usability analysis of the system, their data would be kept anonymously and their observed performance would not have any implications on their professional careers. The study was approved by the METU Human Subjects Research Ethics Committee.

#### Materials and Equipment

**Figure 1:**
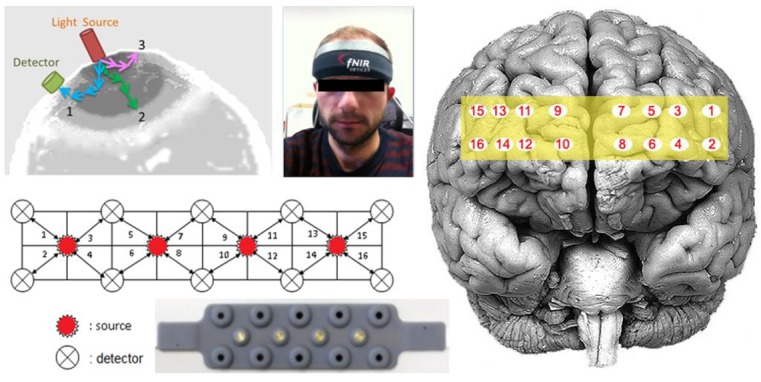
The functional near-infrared spectroscopy (fNIRS) probe with four light sources and 10 detectors, and the corresponding 16 optodes mapped over the prefrontal cortex (PFC).

While the participants were performing the tasks, their eye movements and the hemodynamic changes in their prefrontal cortices were monitored with a mobile eye tracker system (60 Hz, binocular by Pupil Labs GmbH, Germany) and a portable fNIRS system (fNIRS Imager 1002, fNIR Devices LLC, Potomac, MD, USA), respectively. The fNIRS system is composed of a flexible headpiece that holds four LED infrared light sources and 10 photodetectors to obtain oxygenation measures from 16 optodes located over the prefrontal cortex (PFC), a control box for hardware management, and a computer that runs the COBI Studio software (Ayaz et al., 2011) for data acquisition. fNIRS 1002 is a continuous wave NIRS system that uses wavelengths of 730 nm and 850 nm for optical imaging of the PFC. The sensor has a source-detector separation of 2.5 cm, which allows for approximately 1.25 cm penetration depth. This system can monitor changes in relative concentrations of oxy-hemoglobin (HbO) and deoxy-hemoglobin (HbR) at a temporal resolution of 2 Hz. According to co-registration studies that mapped the measurement locations of this fNIRS sensor on a standard brain template (Ayaz et al., 2006; Chen et al., 2017), the 16 optodes correspond to Broadmann areas 9, 10, 44 and 45 (Figure 1). The approximated mapping over a standard brain and the diffuse nature of photon migration paths in the tissue imply that the spatial resolution of the optodes are in the centimeter range (Ayaz et al., 2013).

**Figure 2:**
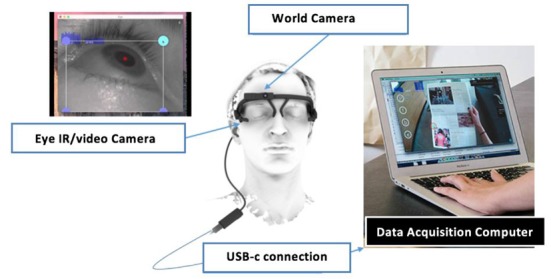
The Pupil Labs mobile eye tracking system.

The eye movements of the participants were monitored with the Pupil Labs mobile eye tracking system. The mobile eye tracker had two infrared cameras and two video cameras facing towards both eyes for binocular recording, as well as an integrated world camera capturing the field of view from the participant’s perspective (Figure 2). The two eye cameras monitor the pupil center and the corneal reflection by utilizing the dark pupil method in reference to a three-dimensional geometric model of the eyeball. The system has a sampling rate of 60 Hz, a reported accuracy of 0.6°, and precision of 0.08°. The external camera can record an external world image at 720 p HD resolution. The PupilCapture open source software accompanied with the mobile eye tracker was used to calibrate and record the eye movements. A nine-point calibration was performed at the beginning of each task and the recorded data was monitored in real time by the researchers for possible indicators of calibration loss or hardware failure.

**Figure 3:**
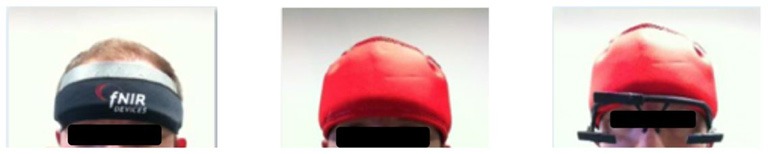
The installation of the fNIRS sensor and the mobile eye tracker for multimodal recording with minimal IR interference.

Since the fNIRS sensor and the eye tracker both rely on infra-red light for the monitoring of brain activity and eye movements, the IR camera of the eye tracker may interfere with the fNIRS signals. Moreover, since the study was conducted as a field study inside a training version of the real platform, the sensors were also partly exposed to sunlight entering through the windows. The fNIRS sensor was shielded with a cap supported with aluminum foil, which can minimize the effects of outside IR sources (Figure 3). Moreover, tests conducted with the eye tracker on and off while recording fNIRS data did not produce any discernable effects on the raw fNIRS signals. This is probably due to the fact that the Pupil Labs system’s IR light source is located beneath the eye balls, which introduces a separation between the eye tracker IR camera and the fNIRS receivers. Due to their location over the forehead and orientation towards the cortex, the LEDs of fNIRS did not introduce any discernable interference on the eye tracking measurements. Excessive sunlight also hampers the eye tracker’s performance and the head-mounted world camera recording the screen, so the built-in roller blinds of the vehicle were used to provide further protective shield from sunlight.

#### Experiment Setup

**Figure 4:**
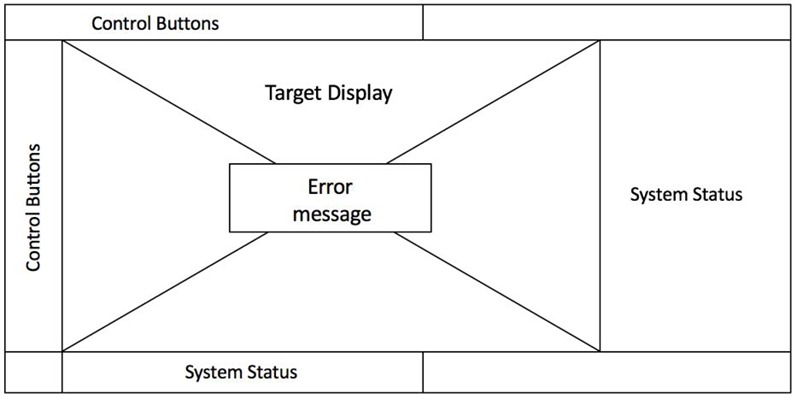
A wirediagram of the graphical user interface used by the participants during the experiment. Error messages were popped up as a separate window in the middle of the screen in the event of exceptions.

During the experiment, participants were seated inside the cabin of a training version of a military land platform designed and manufactured by Roketsan Inc. The training vehicle is an exact replica of the real system with the exception of live ammunition. The operators interacted with the system through a 15.6 inch laptop computer. A wire diagram of the graphical user interface is provided in Figure 4. Each control button on the left opens a window at the center of the screen dedicated to the specific features associated with that function. For instance, pushing the define mission button will bring the target definition screen as a pop-up window in the middle of the screen, which allows the user to either enter coordinates or select a pre-defined target from the list.

#### Procedure

During the experiment, the participants were asked to engage a pre-defined target by using the control interface in normal and adverse conditions. In the first task, participants executed the standard procedure for engaging a target, which required the completion of a sequence of operations including data entry, initialization, system checks, monitoring and target engagement. The second task required participants to engage with a second target in the same way, which was deliberately defined by the researchers as slightly out of range. Briefly after the participants unknowingly selected this target as instructed, the system triggered an unexpected interruption with a system failure message effectively aborting the mission. The translated error message prompted by the system stated “Command Received/Command Aborted: guidance cannot be completed because the cradle’s target information is out of guidance criteria,” which was expected to be challenging for some of the operators to comprehend. Once the system failure message was prompted, the experimenter waited for about 60 s to observe the operator’s response to the prompt, and then verbally asked what might have caused the error. The task was completed when the operator provided an answer. The expected response was related to the fact that the second target was slightly out of range, which was not immediately evident on the system interface.

Although all operators had prior experience with comparable platforms, it was the first time the novice operators worked on the computer-based control interface used in this study. Prior to the experiment, novice operators attended a formal training presentation introducing the basic features of the platform. The experiments were conducted while the novices were using the training platform for the first time. For that reason, novice participants were provided verbal guidance by an expert when they needed while completing the first task. For the second task, participants were asked to follow the regular operation sequence as in the first task. It was observed that novice participants were able to perform the main operations until the error message without the need for guidance as in the first task.

Overall, in line with existing studies on neural and ocular correlates of expertise, we expected experts to elicit less prefrontal activity and exhibit gaze patterns that are better aligned with optimal task sequence due to their familiarity with the system while performing the routine task sequence. In terms of eye movements, we hypothesized that since novices have just been exposed to the environment, they will be searching for the next action in the task sequence, which will require them to inspect more parts of the interface for longer periods of time. We expected this behavior to be reflected in higher saccadic amplitudes and longer fixation durations. For the experts, we expected eye movements to precede the next action with shorter fixation durations and saccadic amplitudes, since the expert is familiar with the task sequence. In the second task, we expected a stronger response from the experts towards the unexpected error when it first appears on the screen, and novices to be less alert since they do not yet have a full grasp of the possible error states and their underlying causes.

## Results

### Behavioral Performance Analysis Findings

**Figure 5:**
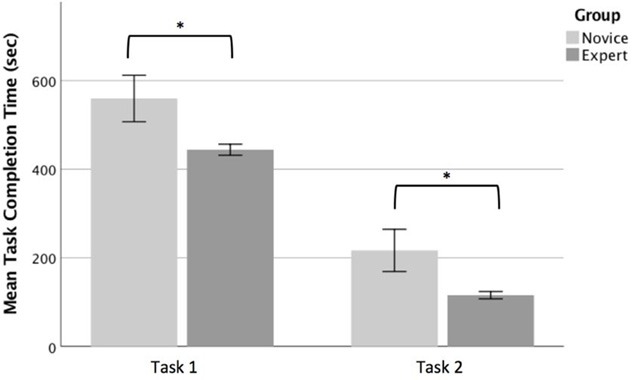
Task completion times for the novice and expert groups for task 1 (left) and task 2 (right). The whiskers indicate standard error. Significant differences are indicated with an asterisk.

During the first task, all participants were able to successfully complete the regular operation sequence. An independent groups *t*-test conducted on the mean task completion times showed that the novices (Mean = 559.7 s, SD = 128.1 s) took significantly longer than the experts (Mean = 444.0 s, SD = 35.1 s), *t*_(12)_ = 2.46, *p* < 0.05 to complete the first task as expected (Figure 5). During the second task, all novice participants failed to identify the cause of the system error, whereas 63% of the expert group was able to correctly interpret the error and provide a satisfactory explanation for the underlying reason. In addition to this, the time to reach the error message (which concluded the task) for the expert participants (Mean = 115.8 s, SD = 23.08 s) was significantly lower than the novice participants (Mean = 216.8 s, SD = 116.8 s), *t*_(12)_ = 2.42, *p* < 0.05 (Figure 5). Although the groups differed in terms of the time to reach the error message, the error was consistently displayed in the same stage following the closing of the target selection screen on the interface. In short, the significant differences in accuracy and task completion times further support our initial grouping of the operators as experts and novices.

### fNIRS Analysis Findings

Filtering of the raw fNIRS data and the conversion to oxygenation measures were carried out by using the fNIR Soft v4.11 (Ayaz, 2010). Firstly, the raw fNIRS data were visually inspected for cases including excessive noise and motion artifacts. Saturated optodes exceeding the signal-to-noise ratio limit of the sensors (if any) were excluded from analysis. The data obtained from one subject was eliminated due to such issues, so the analysis of fNIRS data was conducted over 13 subjects (six novices, seven experts). Sliding Windows Motion Artifact (SMAR) filter (Ayaz, 2010) was used to attenuate signal changes due to head movements. In total, 12 blocks were rejected due to saturation or motion artifacts among the 16 × 13 = 208 blocks, which corresponds to 6% of the collected data. Raw fNIRS data were low-pass filtered with a linear phase filter with order 20 and cut-off frequency 0.1 Hz to reduce the effect of high-frequency noise due to respiration and heart beat cycles. The filtered fNIRS data were then converted into HbO and HbR concentration changes by using the Modified Beer Lambert Law with the default differential path factor and chromophore absorption parameters for adults provided in fNIR Soft. The total hemoglobin (HbT) value, which is defined as the sum of HbO and HbR values observed at each optode and is known to correlate with total cerebral blood flow, was considered as the main dependent variable. The HbT measures were baseline-corrected with respect to the beginning of each block.

#### Task 1–Normal Operation

For the first task, the expert and novice groups were compared in terms of the average HbT levels observed at all optodes. For this purpose, the HbT signals between the beginning and end of the first task were extracted for each participant and block averages were calculated. The differences between the two groups were investigated by using independent *t*-tests conducted for each optode. Overall the average HbT levels tended to be higher for the novices as compared to the experts, especially at optodes 14 and 16 that are close to the right inferior frontal gyrus. However, independent *t*-tests showed that none of these trends reached statistical significance.

#### Task 2–Response to Error Message

For the second task, participants followed an operation sequence similar to the first task. However, the second task included a condition where the system generates an error message at a pre-defined stage. The average HbT values were obtained 20 s before and after the error message was prompted on the participants’ screens. A 2 × 2 mixed analysis of variance (ANOVA) was conducted on average HbT levels to contrast experts and novices in terms of their hemodynamic responses to the error message.

**Figure 6:**
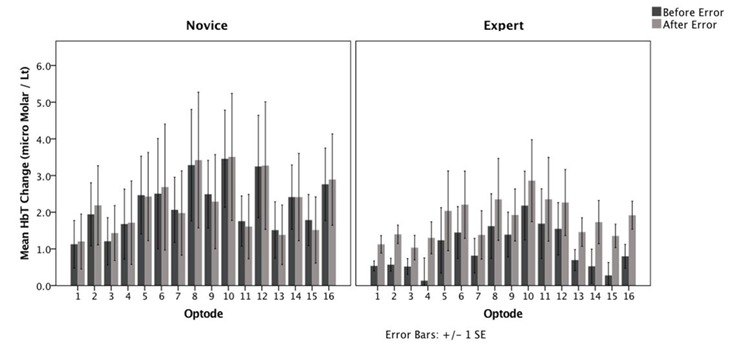
Average HbT changes observed at 16 optodes during the second task before and after the prompted error message.

**Figure 7:**
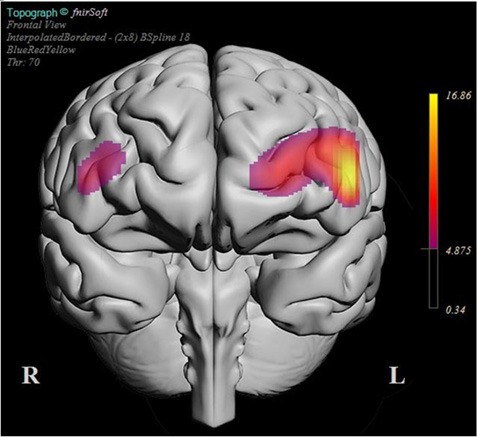
BSpline interpolated F-ratios that exceeded the significance threshold obtained for each optode for the contrast between mean HbT changes during post- and pre-error message. The significant responses in both groups were predominantly localized over the left dorsolateral prefrontal cortex (dlPFC) together with a narrower region within right dlPFC.

**Figure 8:**
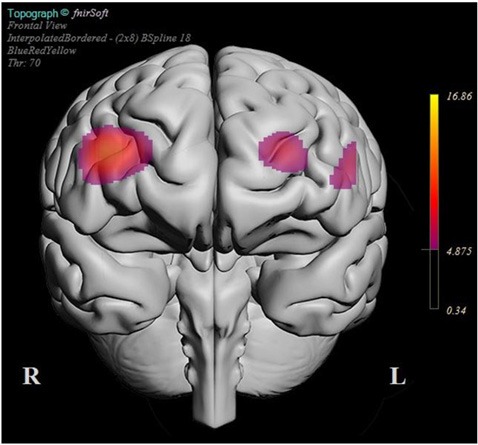
BSpline interpolated F-ratios that exceeded the significance threshold obtained for each optode for the interaction of expertise level and pre-/post-episode on the mean HbT changes. The experts were most distinguished from novices with respect to their HbT response in the right dlPFC when their pre- and post-error average HbT responses were contrasted.

Although the average HbT levels tended to be higher for novices in contrast to experts across all optodes, the main effect of expertise did not reach significance in any one of the 16 optodes, which was consistent with the results obtained for the first task (Figure 6). However, the main effect of pre-/post- distinction and its interaction with expertise levels revealed significant differences, which are summarized in Figures 7, 8, respectively.

The plot in Figure 7 summarizes the locations where significant HbT difference was observed when the post-error block averages were contrasted with the pre-error block averages. The plot is generated in fNIR Soft by using a BSpline interpolation of F-ratios computed for each optode for the main effect of pre-/post-error on mean HbT levels. Only those areas that exceed the statistical significance threshold are highlighted in the plot. The plot suggests that the error prompt elicited the strongest HbT response in the left dlPFC, followed by the left dmPFC and right dlPFC regions for the entire sample. The bilateral dlPFC response is a typical indicator of increased cognitive workload, and the stronger response in the left hemisphere could be interpreted as a reaction to the unanticipated error that aborted the mission.

As shown in Figure 5, the difference between the expert group’s pre- and post-HbT response was overall higher than the novice group. The regions where the interaction between the expertise level and the pre-/post-HbT level differences were significant are shown in Figure 7. The change in the HbT trend in the right dlPFC was particularly stronger in the expert group between pre- and post-error blocks, whereas the novice group exhibited almost no difference in HbT trends in this region. The implicated regions in the right dlPFC are known to be involved with the fronto-parietal network involved with the control of visual attention, so the difference among experts and novices between the levels of pre- and post-error episodes could be related to the attentional demand triggered by the error.

### Eye-Tracking Analysis Findings

Prior to the extraction of gaze measures, the quality of the eye-tracking data was evaluated by going through the video recordings overlaid with raw gaze coordinates. The data of two participants were excluded due to technical issues encountered while finalizing the recorded data with the eye tracker software. Therefore, the analysis of the eye-tracking data was conducted on 12 participants including five novices and seven experts. The velocity-threshold identification (I-VT) filter as implemented by Komogortsev and Karpov (2013) was applied on the raw data to extract the eye fixation durations and the saccadic events. The data obtained from expert and novice operators were compared in terms of their average fixation durations and saccadic amplitudes observed during the first and second tasks.

#### Task 1–Normal Operation

**Figure 9:**
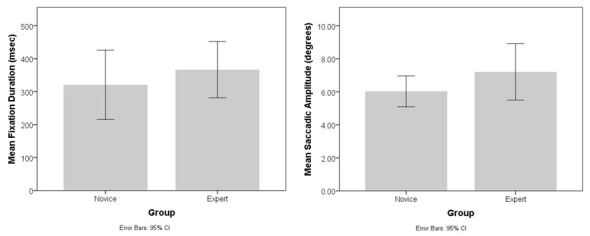
Bar charts showing the average fixation durations (left) and saccadic amplitudes (right) observed for the novice and expert operators during the first task.

During the first task, participants in both groups tended to exhibit similar gaze patterns while following the standard flow on the interface for target engagement. The novice users tended to do more visual search scans to find the relevant menu items for each step of the task, whereas the experts followed a more well-defined gaze sequence directed towards the relevant interface elements. However, no significant difference was observed between expert and novice operators in terms of their mean fixation durations and mean saccadic amplitudes through the course of Task 1 (Figure 9).

We also compared the average fixation duration and saccadic amplitudes during the first and the last minute of Task 1 through 2 × 2 mixed ANOVAs to check if the behavior of the novices and experts differed at different stages of the task. No significant effect of order (*F*_(1,10)_ = 0.43, *p* > 0.05), group (*F*_(1,10)_ = 0.26, *p* > 0.05) and interaction (*F*_(1,10)_ = 0.42, *p* > 0.05) were observed for the average fixation duration measure. A significant main effect of order was observed for the case of saccadic amplitudes, *F*_(1,10)_ = 22.97, *p* < 0.01, partial *η*^2^ = 0.69, but the main effect of group (*F*_(1,10)_ = 0.01, *p* > 0.05) and interaction (*F*_(1,10)_ = 0.45, *p* > 0.05) were not significant. Towards the end of Task 1 both experts and novices had larger average saccadic amplitudes, simply because they had to turn a series of manual switches to finalize the launch that was located towards the left side of the screen.

#### Task 2–Response to Error Message

**Figure 10:**
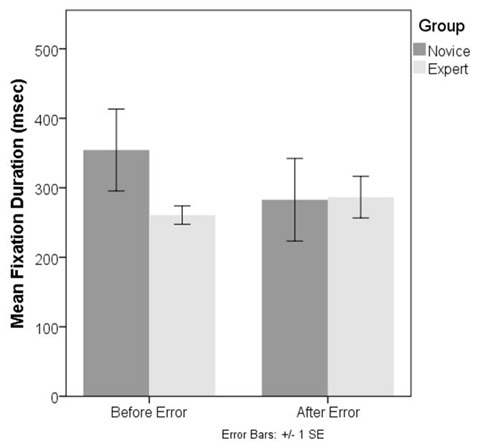
Bar charts showing the average fixation durations observed for the novice and expert operators during the second task.

Similar to the analysis of the fNIRS data, we focused on the influence of the error message on the eye movement patterns of expert and novice operators. For this purpose, the average fixation durations observed for the five fixation events that precede and succeed the error message respectively were compared by using a 2 × 2 mixed ANOVA. The results indicated that the main effect of group and pre-/post- sequence were not significant, whereas the interaction effect was marginally significant, *F*_(1,10)_ = 2.02, *p* = 0.09, one-tailed, partial *η*^2^ = 0.17. The majority of the novices tended to fixate on different parts of the interface for further cues, whereas two novices and one expert (who also could not identify the cause of the error) tended to fixate on the error message for longer durations of time. This led to an unclear pattern where the majority switched into a quick scan mode whereas the others fixated on a single location on the screen, which led to larger variability in the fixation duration measures of the novice participants. On the other hand, those experts who correctly identified the reason tended to switch to a more vigilant mode where they read the message and checked the relevant screen for inspecting the location of the given target. This was accompanied with a slightly increased average fixation duration. However, these qualitative observations did not translate into significant statistical differences in gaze data, possibly due to the small sample size (Figure 10). A similar analysis performed on saccadic amplitudes before and after the error message also did not detect any significant differences.

### Multivariate Analysis Results

**Figure 11:**
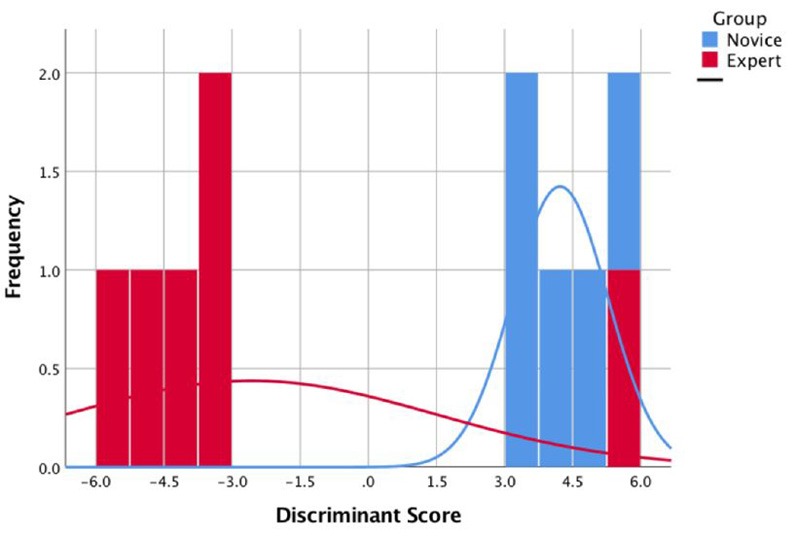
The histogram for the single discriminant function that separate novices (blue) and experts (red).

A multivariate model combining eye-tracking and fNIRS data was also considered based on the findings of our analysis for each modality. The analysis focused on a subsample of 11 participants (five novices, six experts) whose fNIRS and eye-tracking data were complete. Since no meaningful differences could be detected in task 1 in either modality, we focused on task 2 where subjects were prompted with an unexpected error message. A discriminant analysis model was constructed by using the pre- and post-mean HbT changes across 16 optodes and the mean fixation durations as possible predictors in the model. The model that included all the features produced a single discriminant function that failed to significantly differentiate novice and expert groups, Wilk’s *Λ* = 0.019, $\chi_{\left(6\right)}^{2}$ = 11.91, *p* > 0.05, with 64% prediction accuracy in the leave-one-out validation phase. When the model was restricted to those optodes over left and right dlPFC where significant changes in HbT levels were observed for both groups, a significant discrimination was obtained, Wilk’s *Λ* = 0.005, $\chi_{\left(7\right)}^{2}$ = 18.79, *p* < 0.01 with leave-one-out validation accuracy of 73%. The model with the highest validation phase accuracy was obtained when the model was restricted to those optodes in the right dlPFC where we observed a significant interaction effect among pre-post and expert/novice dimensions. In particular, the model with mean HbT levels obtained from optodes 15 and 16 in the right dlPFC and the fixation duration provided a significant discrimination, Wilk’s *Λ* = 0.043, $\chi_{\left(6\right)}^{2}$ = 15.73, *p* < 0.05, with 91% accuracy in leave-one-out validation. The histogram for the single discriminant function estimated by the LDA procedure for this model is shown in Figure 11, where experts and novices were clustered around smaller and lager values, respectively. When we excluded the pre-/post- fixation duration information from the last model and focus only on the HbT features, the validation accuracy dropped to 55%, Wilk’s *Λ* = 0.341, $\chi_{\left(4\right)}^{2}$ = 6.45, *p* > 0.05.

## Discussion

Based on the characterization of neural and ocular correlates of expertise in the related literature, in this study we hypothesized that there would be differences between expert and novice operators in terms of their gaze patterns and prefrontal oxygenation trends while they were engaging with regular and adverse versions of the target engagement task. In particular, since the experts were more familiar with executing the routine target engagement sequence, we expected them to exhibit lower prefrontal oxygenation levels and more structured gaze patterns over relevant parts of the interface as compared to novice operators. In contrast, we expected novices to have more scattered gaze patterns and higher prefrontal oxygenation trends due to the novelty of the interface. Finally, we expected the adverse condition to provide an even stronger contrast between the neurophysiological measures of expert and novice operators.

The findings suggest a less straightforward picture for the normal task condition. Although experts completed both tasks significantly more accurately and more quickly, these behavioral differences were not simply translated into significantly lower prefrontal oxygen consumption and shorter fixation durations. Eye-tracking videos qualitatively suggested that the expert operators were executing the task sequence more smoothly where their gaze patterns tended to precede/anticipate their actions on the interface, whereas the novices exhibited rather a visual search like behavior where they were possibly searching for cues to find the relevant interface elements for the next step. However, none of these qualitative differences translated into significant differences in terms of average fixation duration, saccadic amplitude and prefrontal oxygenation levels during the regular task.

In contrast to the regular task, the adverse task condition turned out to be more effective in terms of revealing significant differences between experts and novices. The unanticipated interruption to the task sequence elicited a significant HbT response at bilateral dlPFC for the experts, whereas there was no change with the exception of a minor, non-significant increase over the left dlPFC for the novices. The interaction effect was most salient over the right dlPFC where experts had a stronger HbT increase following the error, a region known to be part of the right fronto-parietal network related to the management of visual attention. In contrast to hemodynamics effects, univariate analyses of fixation and saccadic amplitudes revealed only a marginally significant interaction effect, which is due to the crisscrossing trends in fixation durations for the experts and novices following the error message. Experts’ fixation durations increased after the error message, whereas the novices’ fixation durations got shorter. A multivariate model exploiting the covariance structure between fixation duration measures and the hemodynamic measures obtained from the right dlPFC could successfully discriminate novices from experts.

The bilateral dlPFC response and elongated fixation durations in the case of experts seem to suggest that experts moved from an automated task processing mode towards a more deliberate attentional state in response to the unanticipated error message. Although we could not monitor the parietal cortex in this study, the overlap between the optodes where we observed a significant interaction and the prefrontal components of the right fronto-parietal attention network seems to support this interpretation.

The case is less clear in the case of novices as their prefrontal responses stayed more or less the same, and they tended to switch to shorter fixation durations. The fact that novices could not provide a reasonable explanation regarding the underlying cause of the error suggests that they had difficulty in interpreting the error message. The obscure language used in the error message itself seemed to have contributed to this issue. Moreover, the error message appeared not immediately after the target was selected, but only a few minutes later while the operator was carrying out the subsequent steps in the launch sequence, which seemed to have contributed to its surprise effect. With the exception of a single operator, the members of the expert group could interpret the meaning of the error message, reportedly based on their past experience with the same problem, whereas novice operators failed to comprehend even whether the error effectively aborted their mission. This highlights the need to consider expertise level and usability aspects hand in hand for cognitive workload assessment.

A key limitation of the study is the small sample size, which is due to the limited availability of the operators and the limited access to the real system for research purposes. Since the system was recently acquired, the population of operators who received training and who were authorized to operate the vehicle were quite limited at the time of this study. This limitation precluded us from running a power analysis to determine the optimal sample size for this study. Although the data set is rather small for a classification analysis, the multivariate discriminant analysis models revealed some key insights regarding the neurophysiological differences between novices and experts when they are presented an unanticipated error. The best classification performance was achieved when HbT averages observed at the right dlPFC were combined with average fixation durations before and after the error message. When we investigated unimodal classifiers the validation accuracy significantly dropped to chance levels. This suggests that a multivariate model tapping on the covariance structure of eye tracking and fNIRS measures may lead to more robust assessments of expertise. In future work, we plan to expand on additional gaze and neuroimaging features, such as gaze entropy and fronto-parietal as well as fronto-temporal connectivity changes, to further develop our understanding of the neurophysiological factors differentiating expert and novice performance in ecological settings.

## Data Availability Statement

The data that support the findings of this study are available from Roketsan Inc. Restrictions apply to the availability of these data since they were collected in a military platform with active personnel. The dataset can be made available with the permission and authorization of Roketsan Inc. and the TAF.

## Ethics Statement

The studies involving human participants were reviewed and approved by Middle East Technical University Human Subjects Ethics Committee. The participants provided their written informed consent to participate in this study.

## Author Contributions

Eİ contributed to the data collection, data cleaning/synchronization, analysis and writing of the manuscript. MÇ contributed to the data collection, fNIRS analysis, multivariate model development and writing of the manuscript. CA contributed to the study design and analysis, interpretation and writing of eye-tracking data. AT contributed to the design and administrative coordination of the study as well as with data interpretation and writing.

## Conflict of Interest

The authors declare that the research was conducted in the absence of any commercial or financial relationships that could be construed as a potential conflict of interest.
